# New Service Virtualisation Approach to Generate the Categorical Fields in the Service Response

**DOI:** 10.3390/s20236776

**Published:** 2020-11-27

**Authors:** Zeinab Farahmandpour, Mehdi Seyedmahmoudian, Alex Stojcevski

**Affiliations:** Faculty of Science, Engineering and Technology, School of Software and Electrical Engineering, Swinburne University of Technology, Melbourne, VIC 3122, Australia; mseyedmahmoudian@swin.edu.au (M.S.); astojcevski@swin.edu.au (A.S.)

**Keywords:** service virtualisation, categorical fields, quality assurance, conditional entropy, joint probability distribution

## Abstract

Software services communicate with different requisite services over the computer network to accomplish their tasks. The requisite services may not be readily available to test a specific service. Thus, service virtualisation has been proposed as an industry solution to ensure availability of the interactive behaviour of the requisite services. However, the existing techniques of virtualisation cannot satisfy the required accuracy or time constraints to keep up with the competitive business world. These constraints sacrifices quality and testing coverage, thereby delaying the delivery of software. We proposed a novel technique to improve the accuracy of the existing service virtualisation solutions without sacrificing time. This method generates the service response and predicts categorical fields in virtualised responses, extending existing research with lower complexity and higher accuracy. The proposed service virtualisation approach uses conditional entropy to identify the fields that can be used to drive the value of each categorical field based on the historical messages. Then, it uses joint probability distribution to find the best values for the categorical fields. The experimental evaluation illustrates that the proposed approach can generate responses with the required fields and accurate values for categorical fields over four data sets with stateful nature.

## 1. Introduction

Currently, enterprise software systems lean towards dividing the software’s task into several subtasks, which are then completed by communication between distributed components named services. Each individual enterprise service, to fulfil its task, may also need to communicate with other outside requisite services from several other vendors [1]. Timely and budget-friendly development of these components with the required quality and performance may be restricted due to some reasons. These reasons may include the imposed access constraint or even substantial cost to the requisite services. These conditions may result in delaying the test of each service until the end of deployment of all services, where any simple fault in one service can cause a chain of faults across multiple services.

Continuous delivery is an industry routine to refine the quality of the software while speeding up the software delivery [2]. These refinements can be achieved by alleviating the integration cost. Thus, each component is tested simultaneously after modification. The components can be continuously tested by ensuring the availability of all their requisite services for a development team.

Multiple methods have been proposed to ensure the availability of the requisite services and the environment to test each service. To imitate the server-side systems’ interactive behaviour, mock objects and stubs [3,4] are commonly used. However, this method requires coding in a specific language; as a result, each service modification leads to the manual change of the code.

Providing a platform in a system to hold several server systems to interact with the component under development, hardware virtualisation or virtual machines tools, such as VirtualBox and VMWare] [5] is proposed and used. This method works with the assumption that the actual requisite server systems are available to be installed on the VMWare or VirtualBox. These systems require actual system resources to be installed; therefore, they cannot scale up easily.

Another solution to set up a much lighter testing environment involves container technologies, such as Docker [6], which provides protected sections of the operating systems instead of virtualising hardware environment. This technology is lightweight; thus, it scales up better than the hardware virtualisation method. However, it has similar limitations for scalability.

Slightly new service emulation approaches [7,8] focus on substituting any requisite services for a component under development with an executable and lightweight estimation. This estimation of the services endeavours to approximate actual service behaviour for particular quality control test and disregard other unnecessary characteristics. Each executable model of a requisite service in the service emulation needs to be determined and configured manually by the experts [7,8]. This approach requires functionality information of each requisite service, which may be unavailable sometimes, especially for requisite service with intricate behaviour.

Service virtualisation (SV) [9] addresses some limitations of service emulation. Firstly, it records the network interaction between a component under development and its requisite services in numeral test scenarios via tools, such as Wireshark [10]. Second, it uses data mining techniques to generate executable models for requisite services [4,9,11,12,13]. The SV solutions are also known as record-and-replay technique because they find the most similar request from the recorded network interactions to the incoming request and replace some of the response fields to generate the new response.

The responses are generated in these SV techniques; they work only in some simple stateless protocols, where a response of a service may only depend on the current request but not on any previous interactions. In addition, they do not work on stateful protocols, where the response values depend on not only the current request but also on the previous interactions. To accommodate the SV for stateful protocols, Enişer and Sen [14] proposed classification-based and machine learning sequence-to-sequence-based methods. However, the presented method requires significant resources and a long time to be trained.

This paper presents cognitive service virtualisation (CSV), a novel software virtualisation method to generate more accurate categorical fields in service response messages. To generate each categorical response field, the technique proposes a new methodical approach, by using mathematical techniques to identify some fields as predictors and use them as clues to find the information in the recorded messages with the highest co-occurrence with the predictor values. The major advantages of the proposed CSV method are highlighted as follows:

- Expanding the record-and-replay method [13] to cater for stateful protocols without sacrificing the time.
- Improving the accuracy of the generated categorical fields [13].
- Considering the long-term dependencies compared with the previous method that cannot accommodate the long-term dependencies [14].
- Significantly less computational power required to generate the accuracy of each categorical fields than state of the art [14].
- Significantly less training time required than the latest method [14].

The remainder of this paper is organised as follows: Section 2 surveys the related work. The problem to be addressed is explained in detail in Section 3. Then, to demonstrate the related work’s challenge in generating categorical fields, we motivate the work with a scenario in Section 4. Section 5 formally presents the structure of the proposed approach. The data sets and the experimental results are presented in Section 6 and Section 7, respectively. Threats to the validity of the reported experiment are discussed in Section 8. The main contributions of the study and future work are summarised in Section 9.

## 2. Related Work

Extracting service interaction behaviour has been an area of focus in several software engineering fields. Therefore, several studies attempted to propose the techniques to mine service behaviour. Amongst those techniques, some SV solutions were proposed to focus on mining the service data and control dependencies from their network interactions.

Cameron [7] proposed an emulation framework tool called Kaluta to substitute service with its runtime execution behaviour model. Kaluta uses the protocol and service specification to generate the interactive service behaviour with service under test (SUT). The generated runtime emulation of the services in this approach is compact and lightweight; therefore, high scalability for testing SUT is achieved.

Several service virtualisation tools, such as IBM [15], HP [16] and Computer Associates [17], have been developed by commercial enterprises. These solutions use service and protocol specifications with some levels of data extracted from service interactions to model their behaviour.

Du proposed opaque SV [18] to decrease human interaction in generating virtualised services. The proposed method focuses on stateless services and uses clustering and sequence alignment to generate the response of each request message, similar to the previously generated ones. These techniques create a virtual service without much prior knowledge of the protocol message format, which was required in most previous solutions. Even though this method is designed for stateless protocols, it cannot address the complex dependencies more than the equivalence between request and corresponding response fields in the stateless protocols as well as any stateful protocol.

To accommodate stateful protocols, Enişer and Sen [14,19] suggested three techniques to generate the responses to the service requests. Their first technique utilised a classification method to incorporate each incoming request and its prior messages to generate each response field’s value. This method assumed the field values as categorical data and utilised a classifier to assign the request and historical messages to one of the values in each response message field. It uses few interactions in the classification without any filter to select the relevant ones. Thus, this technique’s accuracy is limited to situations where all the influential messages are sent and received in a very short window close to the request that is required in generating a response.

The sequence-to-sequence based structure is their second technique, which uses a recurrent neural network (RNN) structure, comprising two LSTM networks. LSTMs are considered useful in sequence-based data sets in several cases, such as language translations [20]. Their RNN structure requires a long time to be trained; therefore, the implementation has been conducted on GPU. In addition, LSTM structures have limitations on the number of historical information they can incorporate.

In the third technique, to generate each categorical response field, they applied a model inference technique (MINT) tool [19]. MINT is a flexible tool designed to infer deterministic guarded extended finite state machines (EFSM) [21]. To infer the guarded variables and merge the states, MINT utilises arbitrary data classifiers. This method has the same limitation as their first technique.

KTail-based method is proposed by Hossain [22] to work on top of opaque SV. The basic idea is to infer the service’s state models to enhance the playback in virtualised services. It has three phases, namely, analysis, model inference and runtime. It defines an event as specific/unique request–response message type pair and event series given that the sequences of events are related to specific service at the analysis phase. Then, at the model inference phase, the state model of each service is inferred from its event sequences by using KTail (K = 0). At runtime phase upon receiving a request, it uses the inferred state model and its current state of the service to specify the response type. Then, it synthesises the response message by dynamic substitution of the nearest matching request type among the interactions. However, this research has improved the state-of-the-art performance of the current service virtualisation (opaque SV), considering the contextual information in generating type of the response message. However, it does not consider the contextual information in generating response contents.

The current service emulation and virtualisation techniques suffer from significant shortcomings. Firstly, Kaluta and commercial SV solutions require considerable developer effort and complex configuration and maintenance. In addition, they require service specifications, which may not be readily available for all services. Secondly, opaque SV works for simple stateless protocols by responding to each request with similar responses in history and some simple substitutions. Thirdly, although the latest published work [14] is designed to work for stateful services, it cannot generate accurate categorical values due to limitations on the number of interactions that it can incorporate to generate each response as well as significant training time. Even though KTail-based method is designed on top of opaque SV to improve its generated response type accuracy, it cannot cover the stateful protocols.

## 3. Problem Statement

Software services communicate with the requisite services by sending and receiving request and responses in a particular amount of time. These exchanged structure of the messages follows the rules of the protocol. The protocol defines that the structure of the information is encoded and transmitted over the network as well as order of the exchanged messages, which are also known as temporal dependencies. Different protocols use different methods to encode the information into messages. Some protocols, such as LDAP, use textual encoding, and others may use binary encoding to transmit the information over a computer network.

Each request received from SUT is assumed to have followed a single response. In situations where the protocol does not follow this assumption, we use the proposed technique by Du et al. [12] to transform messages into request/response pairs.

An appropriate encoder/decoder is assumed to be capable of reading the messages and transforming them into a nonempty sequence of key/value pairs, with each pair containing distinct keys. If repeated keys in a message is allowed in a protocol, then we apply a strategy to convert the repeated keys into distinct keys, such as appending a number to each repeated key.

In addition, we assume that a key exists in all request messages of each protocol that specifies the request type and that we can identify it. This key can be identified by an expert or by using the suggested method by Hossain et al. [23].

Here, we define some of the notations that we use in the rest of the work. In this work, the set of keys are denoted by K. *k* and $k_{i}$ are used to show elements of K. In addition, the set of all values, represented by V. The *v* and $v_{j}$ are used to denote elements of V. We can show a message with *m*, which is a nonempty sequence of key/value pairs. Each key k∈K appears at a maximum of once in *m*. *m* and $m_{k}$ are used to indicate messages. The set of all messages in a protocol is indicated as M. Here, we can formally show a message with the keys $k_{1},k_{2},\ldots k_{n}$ and the corresponding values $v_{1},v_{2},\ldots v_{n}$, respectively, as follows:

$$m\;=\;<k_{1},v_{1}><k_{2},v_{2}>\ldots <k_{n},v_{n}>$$

Each key and its corresponding value <$k_{p}$, $v_{p}$> may also be called a field. Therefore, a message is a nonempty sequence of fields. $m_{q}$ and $m_{p}$ are also used to denote request and response messages, respectively.

In addition, a response message and its corresponding request are also called an interaction. *I*, $I_{i}$ are used to denote individual iterations. For example, $m_{q_{1}}$ and $m_{p_{1}}$ are used to show $I_{1}$ and $m_{q_{i}}$ and $m_{p_{i}}$ are used to show $I_{i}$. Then, the set of interactions is denoted by I. Lastly, a nonempty sequence of interactions $I_{1}$, $I_{2}$, … $I_{j}$ is called a trace and denoted as *T*.

A field’s value can be considered categorical or noncategorical numeric. We assume that a human expert can manually tag each field’s values as categorical or noncategorical numeric. A categorical field can be given one of a confined number of possible values. These values can be numeric or non-numeric such as textual. These numeric values that are provided to those categorical fields are labels and do not follow the same concept and logic as numeric variables. Instead, valid operations for categorical variables are set membership, equivalence and some distribution operations, namely, joint probability distribution. Therefore, we separate categorical fields from the remaining fields. In addition, we presume that the only fields that affect the categorical fields are other categorical fields. Therefore, we only include the categorical fields in the prediction.

We also assume that the initial state in the recorded history transactions can be arbitrary. However, the recorded interactions should be sufficient for the system to determine the state of the requisite service at the end of the final state. Importantly, we assume that the initial state in the test mode is the same as the final state observed in the recorded transactions (training mode). Therefore, the first live request follows the last interaction in the recorded history transactions.

Given the above assumptions and definitions, we represent the service virtualisation problem formally. Given the recorder traces as the sequence of interactions $I_{0}$, $I_{1}$, *…*, $I_{j-1}$ and a live request message $m_{q_{j}}$, a virtual service is required to generate a response $m_{p_{j}}$ that resonates the generated response of an actual service as precisely as possible.

Then, we divide the problem into two subproblems, as follows:

- What keys does a virtual service response require?
- What values of the categorical keys does the virtual service response need to generate?

In the following sections, we aim to detail solutions to these subproblems.

## 4. Motivating Scenario

**Figure 1:**
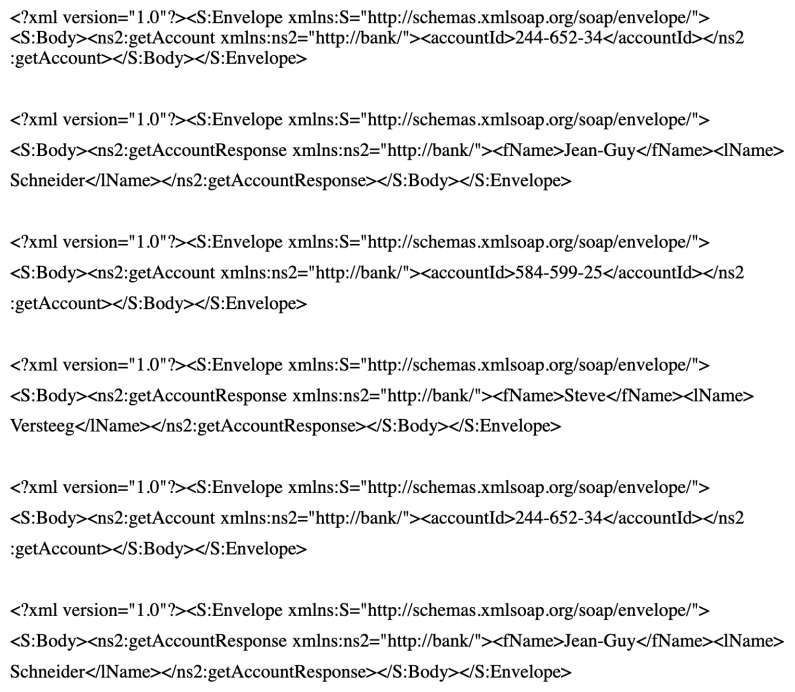
Some interactions from the SOAP banking data set.

To demonstrate the shortcomings of the recent SV solution, such as opaque SV, the requests and their responses in Figure 1 are considered.

With the intention of the following discussion in generating categorical response field, we assume that, in the offline mode, we receive only two requests and their responses as shown in Figure 1. Subsequently, at playback time, we receive an incoming request with *getAccount* type for *accountID* 584-599-25 as follows:

<?xml version="1.0" encoding="UTF-8"?> <S:Envelope xmlns:S="http://schemas.xmlsoap.org/soap/envelope/"><S:Body>

<ns2:getAccount xmlns:ns2="http://bank/"><accountId>584-599-25</accountId></ns2:getAccount> </S:Body></S:Envelope>

Opaque SV [13,18]:

1. Clusters all recorded interactions on the basis of the request message type; in our example, ns2 is the request type field;
2. Organises the same type of interactions into the same cluster and derives a cluster prototype for each cluster;
3. Selects a cluster centre for each cluster; in our example, the interaction includes *accountID* 244-652-34 and *fName Jean-Guy* and *lName Schneider*;
4. Finds the closest cluster centre message of all recorded interactions to the incoming request; in the example, the interaction with *accountID* 244-652-34 and *fName Jean-Guy* and *lName Schneider* is found as the closest match;
5. Identifies fields, which are *symmetric*, indicating the field value repeated in the “closest match” cluster centre request and its response messages; in the example, no symmetric fields exist;
6. Substitutes the symmetric fields’ value in the recorded response by the corresponding symmetric value in the incoming request; in the example, we have the recorded getAccountResponse *fName* and *lName* as *Jean-Guy* and *Schneider* remain the same because no symmetric fields exist.

The generated response message is sent as the response to the incoming request. In this example, the getAccountResponse message in Figure 1 is sent unchanged. Even though this response created to a getAccount request is a getAccountResponse type, which follows the underlying protocol, and the response has an accurate structure; the returned *fName* and *lName* values are inaccurate (i.e., Steve and Versteeg instead of *Jean-Guy* and *Schneider*, respectively). In other words, the generated response contains inaccurate contents.

This examples demonstrate the shortcomings of opaque SV in stateful protocols. In the sections that follow, we present a new technique to deal with such shortcomings.

## 5. Cognitive Service Virtualisation (CSV) Structure

**Figure 2:**
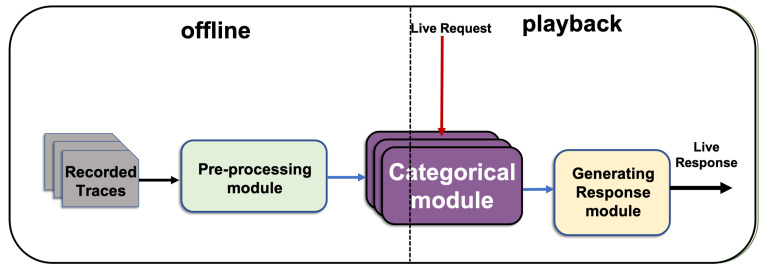
CSV and its components.

At a high level, as shown in Figure 2, CSV has two inputs and one output. Each input and output consists of one or more messages. The system learns from nonempty recorded network interactions between component under the test and the requisite service. This finding is indicated as recorded traces in Figure 2. The second input is the live request message, in which the requisite component is expected to generate a response. The CSV generates a template for the response plus the values of categorical fields. Provided that the recorded traces contain sufficient samples from all request types and their responses, the CSV can generate a live response for any live request. The live response contains all the required keys and their categorical values.

The CSV for each live request message generates a response template (RT) (the anticipated response’s set of keys) on the basis of the relevant messages in the recorded traces and generates the values of the keys identified as categorical. Some key values may contain constants; other values may follow a pattern that depends on the request message type and its contents as well as the relevant recorded messages. If the values of RT depend only on its request message, then the protocol is characterised as stateless. If the values of the RT depends on its request as well as the pertinent prior request and/or response messages, then the protocol is called stateful. The CSV can generate responses for stateless and stateful protocols. It finds the relevant messages from a history of traces and generates the categorical content accordingly.

Figure 2 shows that the CSV consists of three modules, namely preprocessing, categorical and generating response modules. Firstly, in Section 5.1 the preprocessing module processes the recorded traces and separates the unknown categorical fields to be consumed by the categorical module. Secondly, the categorical module in Section 5.2 finds the minimum set of fields that can be used as predictors to best identify the value of each unknown categorical field. Thirdly, the application of the identified predictor to generate the value of each unknown categorical response field is discussed in the generating response module in Section 5.3.

### 5.1. Preprocessing Module

The CSV in the preprocessing module learns how to generate the expected template for a response as well as the constant and symmetric field values.

**Figure 3:**
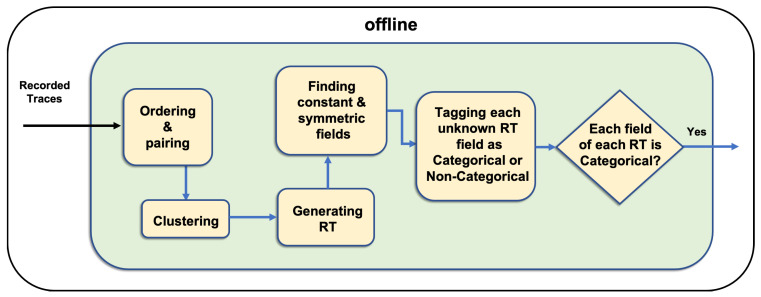
Preprocessing module and its components.

This module works in the offline mode and consists of several components. Thus, the messages are ready for the categorical module [24]. Figure 3 shows that the preprocessing module starts with ordering and pairing component, which assigns an order to the recorded messages by adding an additional key and value such as <messageID,V> to each message in ascending order by using the recorded time. This component also pairs each response and its corresponding request message; this pair is considered an interaction. To generate RT for each request message type, the component initially clusters interactions on the basis of their request message types. Then, in generating RT component a request and a response template out of all request and response message keys for each cluster are generated using the union of all sets of request and response keys. Subsequently, in finding constant and symmetric field component, CSV identifies constant fields, which carry one distinct value across all the messages of the cluster and symmetric fields that contain equal values in each cluster’s request and corresponding response in each RT. It fills the RT with the constant and symmetric values given their availability.

In the next component, it separates unknown categorical fields from the rest to evoke the categorical modules to generate their values. The categorical and noncategorical fields need different methods to be generated. Therefore, the categorical fields are separated to follow the categorical module. Categorical fields (whether numeric or non-numeric) do not require usual mathematical calculations as noncategorical numeric fields to be created. Therefore, we identify and separate the categorical fields. Identifying the categorical fields is done by a user. Categorical fields are assumed to be influenced only by categorical fields. Therefore, other fields (noncategorical) are not incorporated and removed from further processes. In the end of the preprocessing module, each unknown categorical field evokes one instance of the categorical module to generate its value.

### 5.2. Categorical Module

**Figure 4:**
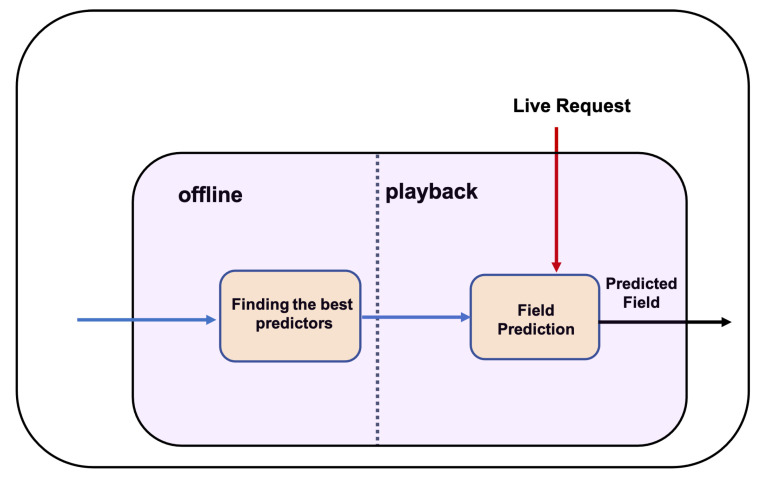
Categorical module.

Each unknown categorical response field evokes one instance of categorical module separately. As shown in Figure 4, the categorical module consists of two modes of offline and playback and two components as finding the best predictors and field prediction. In offline mode, it finds the best set of fields as predictors, and in playback mode, it uses the values of the predictors to identify each categorical field.

#### 5.2.1. Finding the Best Predictors

To find the best predictor/s for each categorical response field, this component finds dependencies amongst different fields in request–response message pairs in the corresponding message type cluster. These dependencies are used to generate the best possible value for categorical fields in each response message. Determining functional dependency between request fields and the categorical response field is possible by finding the best set of fields that can be used to identify the best value. We translate the functional dependencies to a mathematical formula by using the following approach.

A functional dependency A→B indicates that for all instances of a particular value of *A*, *B* always has the same value [25]. Thus, each unique value *A* always determines the unique value of *B*, which is translated to: H(B|A); the variation of *B* given the value of *A* is zero for all unique values of *A*. Therefore, based on the concept of the functional dependency, we found that the conditional entropy concept is similar, and we use it to determine the functional dependencies. Noncategorical fields remarkably vary and cause considerable unnecessary conditional entropy calculations. Therefore, to reduce the unnecessary calculations, we remove noncategorical fields from the set of fields.

Then, by finding the correlation between each request and corresponding response fields using conditional entropy, we identify the best field or combination of fields that can be used as the predictor to generate an accurate value for the unknown categorical response field. We consider all request fields in the request–response pair as candidate predictors and calculate unknown target field’s conditional entropy with other fields. Then, the minimum set of candidate predictors based on conditional entropy is selected as the predictor set of the unknown field. Some of the unknown categorical response fields may not have a predictor to determine their values with 100% accuracy. However, the goal is to find the predictors for each unknown response field from each of their corresponding request fields that generate values more accurately than random assignment of the previously obtained values of the field.

The equation for calculating conditional entropy H(X∣Y) fields X and Y with possible values $\left\{x_{1},\ldots ,x_{n}\right\}$ and $\left\{y_{1},\ldots ,y_{m}\right\}$ and probability function P(Y) is as follows:

(1) $$H\left(X|Y\right)=-\sum_{i,j}P\left(x_{i},y_{j}\right)\log\frac{P(x_{i},y_{j})}{P(y_{j})}$$

As shown in Equation (1), the entropy of field *X* conditioned by field *Y*, considering values $x_{i}$ and $y_{j}$, is calculated. In the equation, $P(x_{i},y_{j})$ is the joint probability of $X=x_{i}$ and $Y=y_{j}$ in the same pair of request–response messages using the Equation (2). $P(y_{j})$ is the probability of occurrence of $y_{j}$ in the messages of that cluster. The summation of the joint probability of each two values of X and Y is multiplied by the logarithm of the division of joint probability of X and Y values on the probability of occurrence of Y values in general.

(2) $$P\left(x_{i},y_{j}\right)=\frac{\mathrm{number}\mathrm{of}\mathrm{pairs}\mathrm{contain}\mathrm{both}x_{i}\mathrm{and}\;y_{j}}{\mathrm{number}\mathrm{of}\mathrm{all}\mathrm{pairs}}$$

Selection of fields as determinants is based on their co-occurrence in an interaction. Thus, correlations of their values are unchecked, except for those that have been already mentioned. The fields are selected as the predictor, where the conditional entropy of the target field conditioned by the predictor is the minimum value among all possible field combinations as candidate predictor, as shown in Equation (3).

(3) $$Predictor=\underset{\begin{array}{c} \mathrm{candidate}\\ \mathrm{predictors} \end{array}}{\mathrm{argmin}}H\left(\mathrm{TargetField}\;|\;\mathrm{candidate}\;\mathrm{candidate}\right)$$

As shown in Equation (3), finding the best predictor that can be a field or a set of fields with minimal conditional entropy requires creating all possible field combinations as candidate predictor and calculating their conditional entropy with the unknown target response field. Finding a combination with the minimum number of fields as the candidate predictor, which has the minimum value for the conditional entropy with the unknown target field among other possible combinations of fields, follows an exhaustive search and is computationally costly. The exhaustive search may be a practical solution for the protocol messages with a very small number of (categorical) fields but seems impractical for protocol messages with any size. Therefore, we propose an approach, which discovers the best possible combination of fields as predictor, without using the exhaustive search with four steps. The proposed four-step approach is as follows:

1. In the request type cluster, given that noncategorical fields remarkably vary and cause excessive unnecessary conditional entropy calculations, we remove noncategorical fields from the set of interaction fields, and the categorical fields are referred to as the candidate predictor set.
2. In the request type cluster, where the target field belongs, one of the fields of the candidate predictor set is selected as the candidate predictor, and the conditional entropy of the unknown target response field conditioned by that selected candidate predictor is calculated using Equation (1). This process is repeated until each field of the predictor set is obtained as a candidate predictor in turn.
3. Then, the candidate predictors of the target response field are sorted on the basis of the value of the calculated conditional entropy in ascending order. Thus, the candidate predictor, which has the lowest conditional entropy amongst other candidate predictors, is considered the best predictor at this stage. In the case of multiple candidate predictors with the same conditional entropy value, they are sorted on the basis of their order of appearance of the field in the request message. Thus, the fields that appear closer to the start of the request message are set as the better single predictors amongst the alternative predictors and stored earlier in the sorted list of predictors in the case of equal conditional entropy values.
4. The first candidate predictor of the sorted list with minimum conditional entropy is considered the predictor. Then, CSV adds the second candidate predictor from the sorted list to the current predictor and considers them as the possible compound predictor. Then, it calculates the conditional entropy of the unknown target response field conditioned by the new possible compound predictors using Equation (1), conditioned on two fields as *Y*. If the value of the recently calculated conditional entropy results in a lower conditional entropy value than the previously calculated conditional entropy, then the new possible compound predictors are assigned as the current predictors. This process of adding another predictor to the set of compound predictor from the sorted list continues until the calculated conditional entropy of the new potential compound predictor set is equal or greater than the conditional entropy of the previous predictor.

#### 5.2.2. Field Prediction

Field prediction component is conducted in playback mode. Playback mode is when the live request comes and the virtual service is expected to respond as the representative of the actual service. On the basis of the generated RT, the categorical fields and their predictors, the system finds the values of the categorical fields using the field prediction component, combines the predicted values in its RT, and sends it as the live response. This component utilises the predictors from the offline mode to generate the value of the categorical field for the incoming live request messages. As shown in Figure 4, to generate each categorical response field, the values of the predictor fields from the live request are used to generate the unknown categorical field. This component uses the identified predictors to find the value for the unknown categorical field with the highest co-occurrence or joint probability with the predictor field values in the recorded traces. To find such a value, the method uses the joint probability distribution of request and response fields using Equation (2) of the training set. The best predictors are used, and the categorical target value may result in more than one value for a particular field. Therefore, the value, which appears in the messages with higher *messageID*, is selected because the message with a higher message *messageID* is more recent.

In addition, the predictor value may not have appeared in the training set; therefore, using predictor’s value cannot create a value for the response field. In that case, one of the values of the response field in the corresponding training cluster type is chosen for the field that has the highest probability of appearing in that corresponding training set cluster.

### 5.3. Generating Response Module

At playback mode, when the virtualised service receives live requests, its message type field is used to select the corresponding RT, constant and some of the symmetric fields, where their symmetric pairs are known. Then, the categorical fields of the selected RT are generated on the basis of the availability of the identified predictors’ values.

## 6. Data Set Description

Four data sets are used to evaluate the CSV namely, first banking, second banking, calculator and LDAP data sets. In actual service virtualisation scenarios, limited training data are provided by the clients to the companies to generate virtual services. This limitation from the companies is totally understandable due to the privacy purposes of their service users. Therefore, in case of simulating data sets, we simulate them as close as possible to the actual scenarios and test the CSV to show its effectiveness in a “real-world” problem and situation. Starting with two banking data sets with different scenarios, which contain the network interactions between an automated teller machine (ATM) and its banking server. Obtaining the actual transactions between an ATM and the banking server is infeasible due to the security consideration. Therefore, we generated them to be as close as possible to the actual transactions. Then, the calculator and the third generated data set are explained, thereby emulating the interactions between a client and calculator API. The forth and the last data set is the recorded transaction between the LDAP client and its server which was provided by the industry partner for our evaluations. The data sets used in our evaluation are explained in detail in the following sections.

### 6.1. Banking Data Set

The banking data set comprises network interactions between an ATM and the banking server using SOAP protocol. To test the ATM service, the banking server needs to be virtualised in such a way that it mimics the real logic and behaviour of the banking server without any knowledge of the protocol or the servers’ behaviour. In an actual scenario, ATM needs to communicate with the server, deposit to, withdraw from and obtain the balance values and account information of the specific bank account by using different message types.

We created two data sets, each with different scenarios and sets of transactions, to be similar to the real-world scenario. The first one consists of small number of interactions to evaluate CSV in the face of small data sets, which relatively reflects the actual situation where the solution is required, to work in scenarios, where data available are insufficient. The second data set contains higher number of interactions and follows slightly different scenario to evaluate the effect of changes in the scenario on the CSV, as explained in the subsequent section.

#### 6.1.1. First Banking Data Set

The generated banking data set comprises simple noninterleaving traces, where each trace only includes the transactions related to one account ID. The banking data set includes six types of request messages including *getNewToken*, *depositMoney*, *withdrawMoney*, *getAccount*, *getTransactions*, *deleteToken* and their corresponding responses as *getNewTokenResponse*, *depositMoneyResponse*, *withdrawMoneyResponse*, *getAccountResponse*, *getTransactionsResponse*, *deleteTokenResponse*, respectively. The *getNewToken* request message requests the new trace, which is responded with the specific token value related to one specific account ID, and *deleteToken* marks the end of that specific trace with the specific token. The *depositMoney* deposits money to the specified account ID in the request message, and *withdrawMoney* withdraws money to the specified account ID. The *getAccount* requests the first name and the last name of the account owner for the specified account ID mentioned in its request. The *getTransactions* returns the exchanged transactions, where each account ID contains the same values in the response.

**Figure 5:**
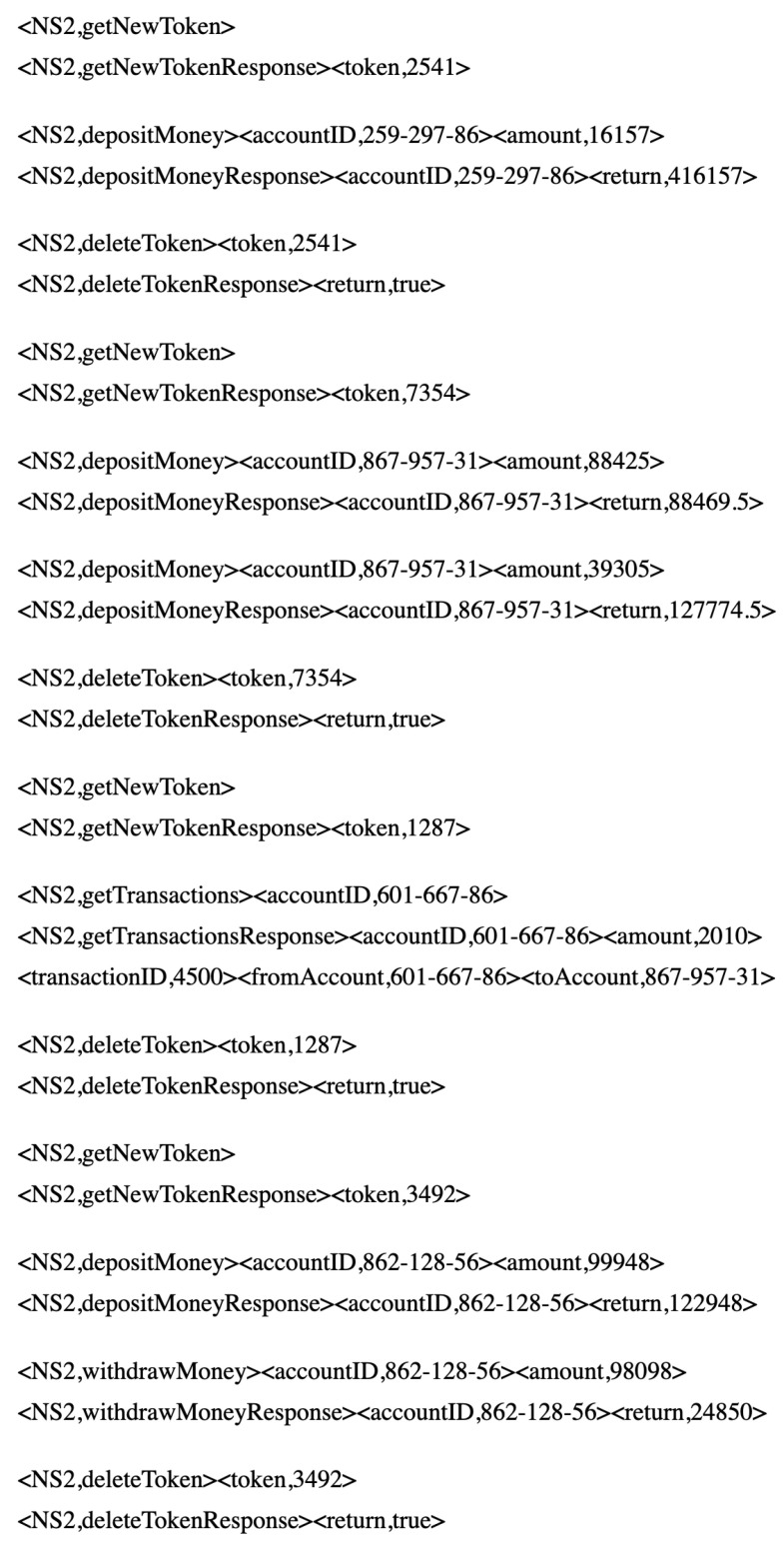
First banking data set samples [24].

The first banking data set contains 300 traces; each trace may contain the interactions related to one out of 10 account IDs. Each trace length varies between three and eight messages. The data set has 1141 pairs of request and corresponding response messages. This data set includes six types of request messages. Each trace is started with getNewToken and ended with deleteToken. Therefore, these message types appear exactly once in each trace. Then, [0–8] instances of messages of types getAccount, depositMoney, withdrawMoney and getTransactions can be found. Here, the first banking data set samples are shown in Figure 5.

#### 6.1.2. Second Banking Data Set

The second data set contains 3000 traces and 29,715 sets of interactions, including seven different types of request messages, such as *getNewToken*, *depositMoney*, *withdrawMoney*, *getBalance*, *getTransaction*, *getAccount*, *deleteToken*, followed by their corresponding response types of *getNewTokenResponse*, *depositMoneyResponse*, *withdrawMoneyResponse*, *getBalanceResponse*, *getTransactionResponse*, *getAccountResponse*, *deleteTokenResponse*, respectively. The second data set has one more request message type as getBalance and its corresponding response getBalanceResponse than the first one. In addition, the response messages of withdrawMoney and depositMoney do not contain account balances. However, the account balance can be requested by getBalance request message, followed by its corresponding response.

**Figure 6:**
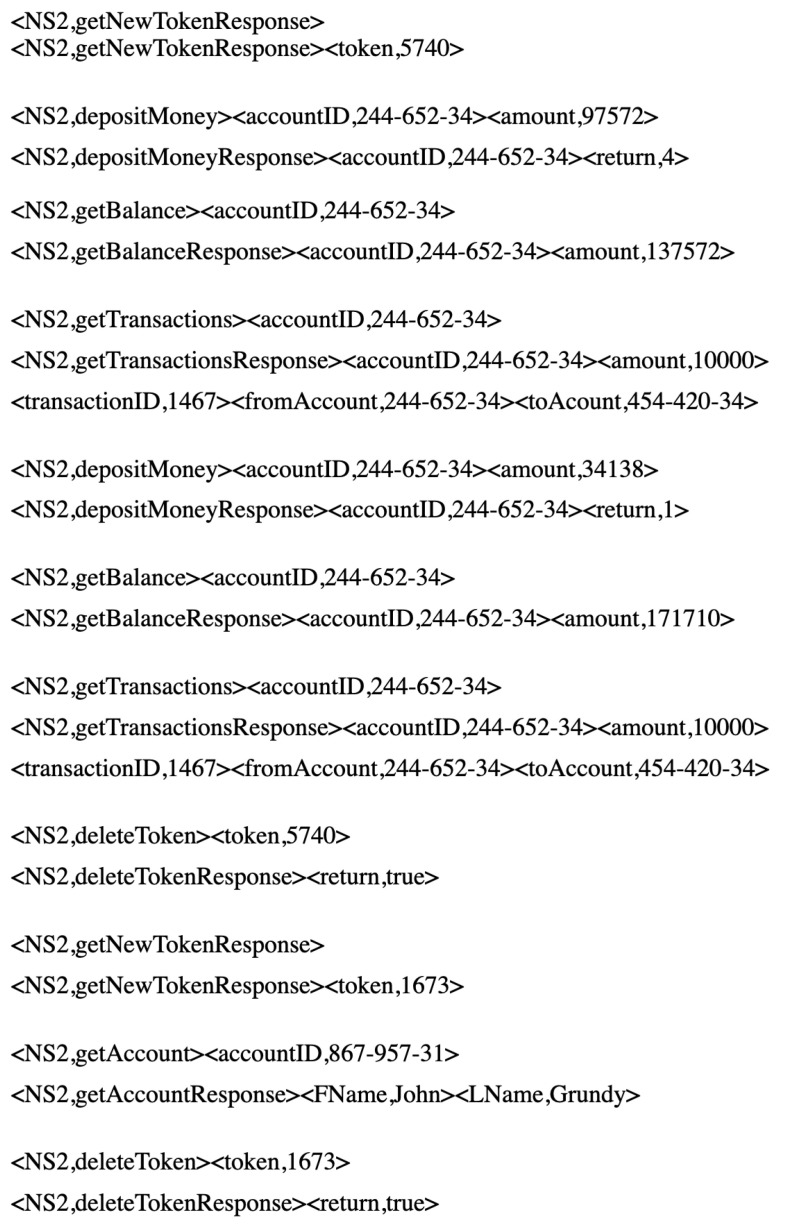
Second banking data set samples.

In an actual scenario, the ATM users have greater tendencies to check the balance of their account after deposit and withdrawal transactions. However, user transactions are not only limited to deposits and withdrawals. Therefore, to generate a data set similar to traces of an actual ATM, the getBalance request message is followed with a probability of 60% after each withdrawMoney and depositMoney transaction. Then, the assumption that not all ATM users determine their account balances after withdrawal or deposit transactions is considered. Here, some sample messages from the second data set are shown in Figure 6.

### 6.2. Calculator Data Set

**Figure 7:**
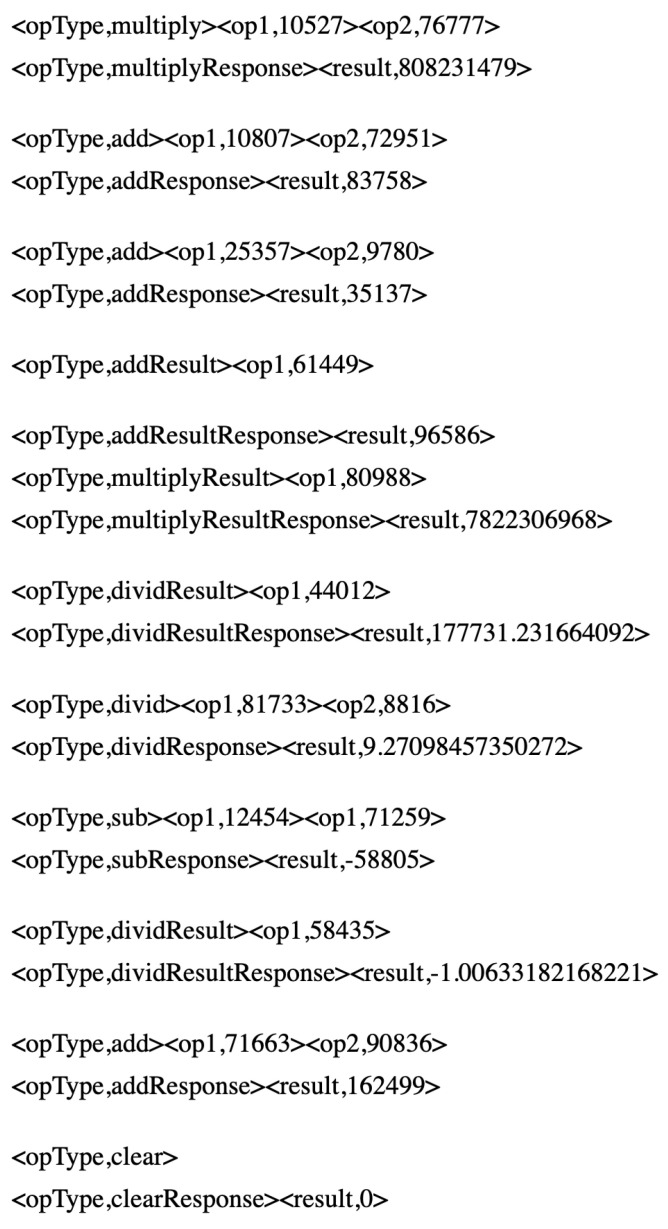
Calculator data set samples [24].

The calculator data set emulates the network transactions between a client and the calculator API. It contains 5000 interactions equivalent to 10,000 messages with eight request message types, as clear, add, addResult, subtract, multiply, subResult, multiplyResult and divide. Each request message type is followed by its corresponding response message. The data set has approximately equal chance of determining each request type. This data set contains simple noninterleaving network transactions. A response with a zero result follows the clear request message. Messages with two operands are multiply, divid, add and sub. Each request types is followed by its corresponding response message with the result of the specified operation on its two operands. Message types with one operand in their requests are multiplyResult, subResult and addResult. Each operand request type follows a response with the result of its operation on their only operand and the last response result value. The calculator data set samples are shown in Figure 7.

### 6.3. Ldap Data Set

The Lightweight Directory Access Protocol (LDAP), is a versatile and standards-based protocol for communicating with directory servers [26]. It is applied for authentication and recording information. LDAP data set is a set of real recorded transactions between an LDAP server and its client provided by the industry partner. It contains 4265 messages with different types namely， *LDAP Bind Request*, *LDAP Bind Response*, *LDAP Add Request*, *LDAP Add Response*, LDAP Compare Request, *LDAP Compare Response*, LDAP Delete Request, *LDAP Delete Response*, LDAP Modify DN Request, *LDAP Modify DN Response*, LDAP Modify Request and *LDAP Modify Response*, LDAP Search Request, *LDAP Search Result Done*, LDAP Search Result Entry and *LDAP Unbind Request*.

**Figure 8:**
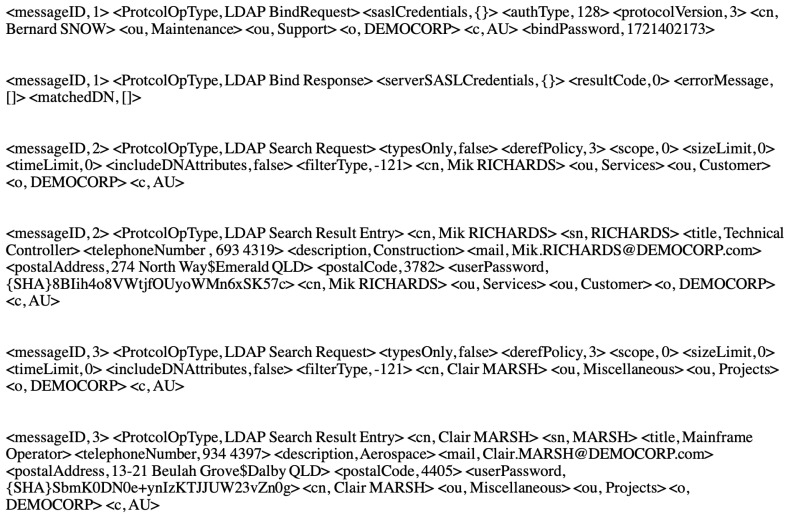
LDAP data set samples.

*Each session starts with one LDAP Bind Request and its response as LDAP Bind Response and ends with an* LDAP Unbind Request. Meanwhile, any of the other requests and their corresponding response types can appear. Here, some sample messages from the LDAP data set are shown in Figure 8.

## 7. Evaluation

We start the evaluation process by comparing our approach with several methods on the basis of different benchmarks in Section 7.1. Then, we select one technique and compare the evaluation result of this method with the proposed CSV in Section 7.2. Subsequently, the section is followed by a detailed analysis of the results.

### 7.1. Comparison Benchmarks

In this section, the state-of-the-art SV solutions are compared with the proposed CSV method on nine different benchmarks, namely, accuracy, reliability, required data set size, computational cost, training time, automation, complexity, robustness and dependency coverage. Accuracy is the degree that the generated response message from the virtual service resembles the response from the actual service. In addition, reliability is the degree to which the results perform consistently well in several protocols with different structures and properties. Robustness is the ability to withstand adverse conditions or rigorous testing throughout different protocols. Table 1 shows the comparison between five different service virtualisation methods, namely, opaque SV [13], LSTM-based method [19], KTail-based method [22], commercial SVs [15,16,17] and the proposed CSV. These methods are compared using nine different benchmarks.

The LSTM-based method, commercial SV and CSV, gain high accuracy, contrary to opaque SV and KTail-based methods. All four methods are relatively reliable; however, the commercial SV is highly reliable because it is generated using protocol and service specifications. The required data set size of all five methods to gain accuracies are shown. Opaque SV does not require a large data set to generate the virtual service with the mentioned accuracy. Commercial SV solutions mostly use the service and protocol specifications and data sets to find some alternative options for the fields; therefore, they only require a small data set. The proposed CSV requires low- to medium-size data set. On the contrary, KTail-based method requires a large data set to generate the reliable FSM. LSTM-based method also requires a large data set to learn to replicate the response messages.

The computational cost for opaque SV, which uses sequence alignment techniques and works on the byte level information of the messages, is medium. The computational cost of CSV and KTail-based methods is also medium as well. LSTM networks are designed to address the vanishing gradient problem of RNN, which helps to remember previous data in memory [27]. Commercial SVs have a low-to-medium computational cost, depending on the service and/or protocol complexity. LSTM uses multiple switch gates and a sequential path from previous cells to the current one to learn longer-term information. However, the time required for the LSTM network consider the old information is limited. In addition, LSTM applies back-propagation to train the model, and a normal system requires a long time to be trained because it has a sequential structure. LSTM-based solution has a very high computational cost and very long training time, which may take days on a normal system, whereas the other methods require a short time to train (in terms of minutes).

From the automation point of view, opaque SV and KTail-based methods could be considered semiautomatic with a level of user interference. LSTM-based and the proposed CSV require very little user interference and are considered mostly automatic. On the contrary, commercial SVs require an expert to manually code the service and protocol characteristics, viewed mostly as manual. The complexity of the methods could be crucial when the method requires any level of human intervention. The proposed CSV, opaque SV and KTail-based methods are not complex. However, LSTM-based and commercial SVs could be complex.

**Figure 9:**
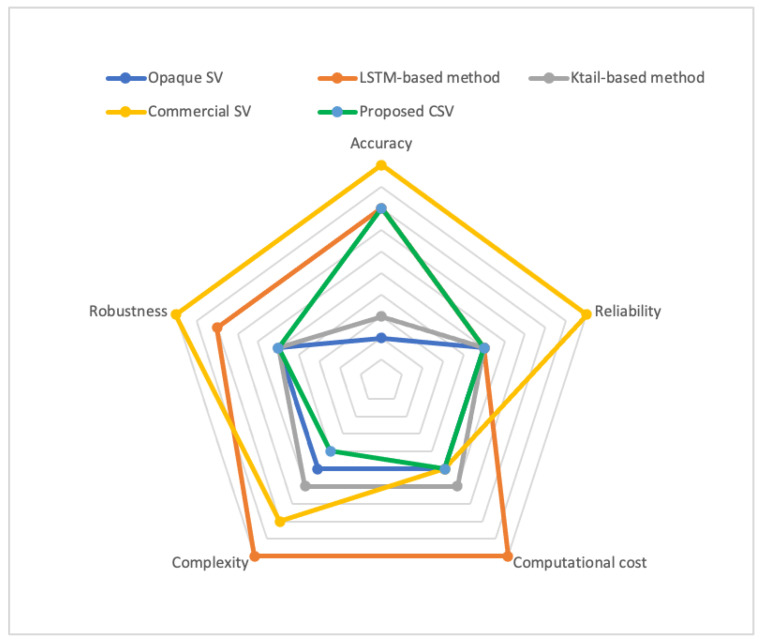
Comparison of proposed technique with state-of-the-art methods according to five benchmarks.

Moreover, due to the variety of protocols and security requirements, they may require a different level of decryption methods prior to virtualisation. Therefore, no automatic systems can deal with decrypted protocols; thus, low robustness is obtained. However, because the commercial SV solutions use the service specifications, they can handle all the necessary decryption levels and have high robustness. The proposed CSV and the commercial SV can incorporate long-term dependencies, whereas the other methods only cover short-term dependencies. Figure 9 visually compares the proposed CSV method with state-of-the-art methods on five main benchmarks, namely, accuracy, reliability, computational cost, complexity and robustness.

### 7.2. Data Sets Evaluation Results

**Figure 10:**
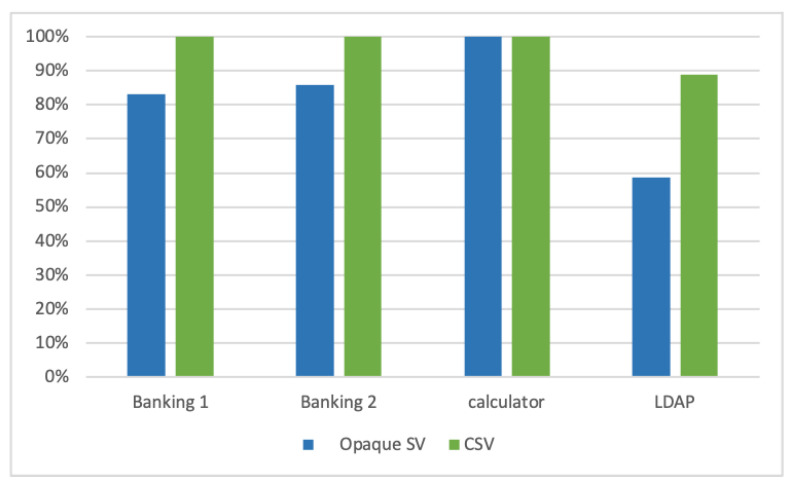
Accuracy on generated categorical fields between opaque SV and CSV on four data sets.

We compared CSV with four different recent works on the basis of several benchmarks. Amongst them, only opaque SV provides a viable and practical solution, which is used to address the actual problem in the industry, due to some limitations of the other SV solutions. Therefore, in this section, we select opaque SV to compare with the proposed solution quantitatively. Figure 10 shows the comparison between opaque SV and CSV results whilst generating categorical fields on the first banking, second banking, calculator and LDAP data sets. The first 90% of each data set are used as training set and the last 10% as test set. Here, accuracy is the ratio of accurately generated categorical values in the response messages to the number of all categorical values in all generated responses. The comparison chart shows that CSV obtains 100%, 100%, 100% and 88.7% accurate categorical fields for first banking, second banking, calculator and LDAP data sets, respectively. However, opaque SV can generate 100% accurate results for calculator data set, which contains only constant fields while generating 83%, 86% and 58.6% accurate categorical fields on the second, third and LDAP data sets, respectively.

In a more detailed analysis of the generated categorical fields on each data set per message type, we start with the first and second banking data sets. We compare the result of CSV and opaque SV on different message types in Table 2 and Table 3. The table shows that all message types have one common categorical fields in their response messages. The field is NS2, which contains equal and constant values, which are accurately generated by CSV and opaque SV. The response messages contain the same accountID value as the corresponding response accountID. Therefore, CSV easily finds them as symmetric values in the component mentioned in Section 5.1. Opaque SV uses byte sequence alignment between request and response messages. Therefore, it finds the request accountID and its corresponding response accountID as symmetric values and generates it accurately.

As shown in Table 2 and Table 3, the difference between CSV and opaque SV in generating categorical fields is the ability of CSV in generating the accurate values for transactionID, fromAccount, toAccount, FName and LName. The getTransaction response type returns the information about the last interaction, which are considered constant values separately for each accountID across all interactions in both data sets. On the contrary, getAccount response returns the first name and the last name of the account owner in FName and LName values. In getAccount and getTransactions message types, CSV identified accountID field as the best predictor for the categorical response fields and used the value of getAccount as the indicator to find the values of FName, LName, transactionID, fromAccount and toAccount; however, opaque SV failed.

The results of CSV and opaque SV on generating categorical values of the calculator data set are shown in Table 4. All message types have one categorical field as opType in their response, which has a constant value across all messages of each type; therefore, CSV and opaque SV generated them accurately.

Table 5 shows each categorical response field and its prediction accuracy across different message types for both CSV and opaque SV methods. *LDAP Search Result Done* and *LDAP Add Response* only contain keys with constant values; therefore, both methods accurately generate their values. All other message types contain both constant and variable response messages. This data set contains categorical response fields with either constant values or variable. *messageID* is a symmetric field which appears across all the request and response types. Both CSV and opaque SV generate the values of all response *messageID*s accurately. Other than *messageID* s, opaque SV could only generate the accurate values of fields with constant values which are shown with 100% accuracy in the last column of Table 5. Opaque SV failed to generate accurate values for the rest of fields which are neither constant nor symmetric. On the contrary, the proposed CSV generates the values of them with accuracies mentioned in the third column of the table. As shown in Table 5 shows, the proposed CSV significantly improves the accuracy of generating categorical fields’ values in the LDAP data set.

## 8. Threats to Validity

The system’s state can be identified using the reflected information in the recorded transactions. Therefore, for the CSV to work accurately, a complete set of relevant messages needs to be available for the system. The training as well as test data set for the CSV have some constraints. The CSV learns models per each categorical response field in each request type cluster. Therefore, to train the data set, it should contain all the message types, which are provided at playback mode as the test set. The current version of CSV cannot generate a response to the request types that do not exist in the recorded traces. In addition, in scenarios with stateful nature, the messages provided to the virtual system at playback mode should have related interactions in the recorded training data to infer their states. In addition, the current version of CSV cannot address the situations, where categorical information in one message type is altered by other types. Therefore, expanding the CSV to incorporate different message types in generating the categorical response fields can be addressed in the future work.

## 9. Conclusions and Future Work

This paper presents CSV as a novel approach for generating the values of categorical response fields. This approach is proposed to improve the state-of-the-art SV solutions in terms of accuracy. The CSV works in two modes of offline and playback. In offline mode, the categorical module finds the best set of fields in each request type that can be used to predict the value of the unknown categorical response field. Thus, it finds the minimum number of fields that have minimum conditional entropy with the target fields as predictors. Using conditional entropy for that purpose can measure the variation of the categorical target response field by obtaining the value of other candidate predictors. By using conditional entropy to measure the target field’s variation, the method finds a set of fields. Thus, identifying their values could obtain the lowest variation of values in the specific categorical response field and call them predictors. In the playback mode, the value of the predictor fields from the live request is used to search the interactions in all the type training set, and the value for the unknown categorical field with the highest co-occurrence with the predictor field values is identified. CSV has been compared with opaque SV, and a promising result on the test data sets is achieved.

This study provides an automated and more accurate virtual services, which consequently could be used in several different test scenarios. The implication of this research could assist continuous testing and accordingly facilitate the continuous delivery of software services. Despite the promising result illustrated at obtaining categorical values for the response messages, a number of other issues remained. In the future, we plan to work on two prime issues, as follows: (i) extending the proposed technique to a framework to work with most protocols effectively, and (ii) expanding existing work to incorporate different message types’ influence in generating categorical fields. In addition, the technique needs to be verified on more data sets with different dependencies and service behaviour beyond the current examined data sets.

## Figures and Tables

**Table 1:** Comparison between Opaque SV, LSTM-based and KTail-based methods, commercial SV and the proposed SV.

Methods	Opaque SV [13]	LSTM- Based Method [19]	KTail-Based Method [22]	Commercial SV [15,16,17]	Proposed CSV
Benchmarks					
Accuracy	low	high	low	high	high
Reliability	medium	medium	medium	high	medium
Data set size	small	large	large	small	small-medium
Computational cost	medium	very high	medium	low-medium	medium
Training time	short	long	short	long	short
automation	semi	mostly	semi	mostly	mostly
	automatic	automatic	automatic	manual	automatic
Complexity	low	high	low	high	low
Robustness	low	high	low	high	low
Dependency coverage	short	short	short	long	long
	term	term	term	term	term

**Table 2:** Detailed comparison of generated categorical fields by CSV and opaque SV on the first banking data set across different message types.

Response Message Type	Categorical Fields	Accuracy of CSV	Accuracy of Opaque SV
getNewToken	NS2	100%	100%
getAccount	NS2	100%	100%
	accountID	100%	100%
	transactionID	100%	0%
	fromAccount	100%	0%
	toAccount	100%	0%
getAccount	NS2	100%	100%
	FName	100%	0%
	LName	100%	0%
withdrawMoney	NS2	100%	100%
	accountID	100%	100%
depositMoney	NS2	100%	100%
	accountID	100%	100%
deleteToken	NS2	100%	100%
	return	100%	100%

**Table 3:** Detailed comparison of categorical fields generated by CSV and opaque SV on the second banking data set across different message types.

Response Message Type	Categorical Fields	Accuracy of CSV	Accuracy of Opaque SV
getNewToken	NS2	100%	100%
getTransactions	NS2	100%	100%
	accountID	100%	100%
	transactionID	100%	0%
	fromAccount	100%	0%
	toAccount	100%	0%
getAccount	NS2	100%	100%
	FName	100%	0%
	LName	100%	0%
withdrawMoney	NS2	100%	100%
	accountID	100%	100%
depositMoney	NS2	100%	100%
	accountID	100%	100%
getBalance	NS2	100%	100%
	accountID	100%	100%
deleteToken	NS2	100%	100%
	return	100%	100%

**Table 4:** Detailed comparison between CSV and opaque SV on generating categorical fields of the calculator data set across different message types.

Response Message Type	Categorical Field	Accuracy of CSV	Accuracy of Opaque SV
Clear	opType	100%	100%
add	opType	100%	100%
addResult	opType	100%	100%
sub	opType	100%	100%
subResult	opType	100%	100%
multiply	opType	100%	100%
multiplyResult	opType	100%	100%
divide	opType	100%	100%
divideResult	opType	100%	100%

**Table 5:** Detailed comparison between CSV and opaque SV on generating categorical fields of LDAP data set across different message types.

Response Message Type	Categorical Field	Accuracy of CSV	Accuracy of Opaque SV
LDAP Bind Response	resultCode	91%	0%
	messageID, ProtcolOpType,	100%	100%
	serverSASLCredentials,	100%	100%
	errorMessage, matchedDN	100%	100%
LDAP Modify Response	resultCode, errorMessage	100%	0%
	messageID, ProtcolOpType	100%	100%
	matchedDN	100%	100%
LDAP Modify DN Response	resultCode	83%	0%
	ou, ou, o, c, matchedDN	83%	0%
	messageID, ProtcolOpType	100%	100%
	errorMessage	100%	100%
LDAP Delete Response	resultCode	60%	0%
	ou, ou, o, c	60%	0%
	matchedDN	60%	0%
	messageID, ProtcolOpType	100%	100%
LDAP Compare Response	resultCode	100%	0%
	matchedDN, c, o	100%	0%
	messageID, ProtcolOpType	100%	100%
	errorMessage	100%	100%
LDAP Search Result Entry	cn	24%	0%
	sn	57%	0%
	ou	12%	0%
	ou, title, description	30%	0%
	mail, userPassword	30%	0%
	telephoneNumber	43%	0%
	postalAddress, postalCode	39%	0%
	mobile	81%	0%
	messageID, ProtcolOpType, o, c	100%	100%
LDAP Search Result Done	messageID, ProtcolOpType	100%	100%
	resultCode, errorMessage	100%	100%
	matchedDN	100%	100%
LDAP Add Response	messageID, ProtcolOpType	100%	100%
	resultCode, matchedDN	100%	100%
